# Automated inflammatory bowel disease detection using wearable bowel sound event spotting

**DOI:** 10.3389/fdgth.2025.1514757

**Published:** 2025-03-13

**Authors:** Annalisa Baronetto, Sarah Fischer, Markus F. Neurath, Oliver Amft

**Affiliations:** ^1^ Hahn-Schickard, Freiburg, Germany; ^2^ Intelligent Embedded Systems Lab, University of Freiburg, Freiburg, Germany; ^3^ Medical Clinic 1, University Hospital Erlangen, Friedrich-Alexander Universität Erlangen-Nürnberg, Erlangen, Germany; ^4^ Deutsches Zentrum Immuntherapie, Erlangen, Germany

**Keywords:** bowel sound, machine learning, ubiquitous computing, digestion monitoring, inflammatory bowel disease

## Abstract

**Introduction:**

Inflammatory bowel disorders may result in abnormal Bowel Sound (BS) characteristics during auscultation. We employ pattern spotting to detect rare bowel BS events in continuous abdominal recordings using a smart T-shirt with embedded miniaturised microphones. Subsequently, we investigate the clinical relevance of BS spotting in a classification task to distinguish patients diagnosed with inflammatory bowel disease (IBD) and healthy controls.

**Methods:**

Abdominal recordings were obtained from 24 patients with IBD with varying disease activity and 21 healthy controls across different digestive phases. In total, approximately 281 h of audio data were inspected by expert raters and thereof 136 h were manually annotated for BS events. A deep-learning-based audio pattern spotting algorithm was trained to retrieve BS events. Subsequently, features were extracted around detected BS events and a Gradient Boosting Classifier was trained to classify patients with IBD vs. healthy controls. We further explored classification window size, feature relevance, and the link between BS-based IBD classification performance and IBD activity.

**Results:**

Stratified group K-fold cross-validation experiments yielded a mean area under the receiver operating characteristic curve ≥0.83 regardless of whether BS were manually annotated or detected by the BS spotting algorithm.

**Discussion:**

Automated BS retrieval and our BS event classification approach have the potential to support diagnosis and treatment of patients with IBD.

## 1 Introduction

Inflammatory bowel diseases (IBD) are chronic conditions that can have an impact on patients’ quality of life. The two main forms of IBD, ulcerative colitis (UC) and Chron’s disease (CD), cause inflammation at specific locations of the gastrointestinal (GI) tract, resulting in symptoms including abdominal pain, diarrhoea, rectal bleeding, and weight loss (1). Patients with IBD experience periods of disease remission and relapse and possibly require frequent hospitalisation. Overall, it was estimated that 0.3% of the European population and 0.5% of the North American population were diagnosed with IBD (2). While IBD is mostly prevalent in Western countries, recent studies reported increasing incidence in Eastern and developing countries (2).

To determine IBD conditions, various diagnostic investigations are recommended (3). Non-invasive assessment includes anamnesis, physical examination, and bowel ultrasound. GI inflammation should further be examined by analysing relevant biomarkers collected from blood and stool samples (3). However, none of the available biomarkers are IBD specific, therefore guidelines recommend an endoscopic evaluation at the early stage of the diagnosis (3). Endoscopic procedures require bowel preparation, cause patient discomfort, and are expensive (3, 4). Current IBD diagnosis relies on a combination of clinical disease features, endoscopy, non-invasive imaging, and biomarkers of inflammation, which possibly makes IBD diagnosis challenging.

Over the last decades, Artificial Intelligence (AI)-based methods were proposed to support IBD diagnosis and treatment. Various AI-based models were tested to diagnose as well as predict risk of IBD (5). For example, Han et al. (6) analysed the performance of a Random Forest Classifier in detecting IBD using features extracted from RNA expression data. On a dataset including 163 samples from patients with IBD and 109 samples from healthy controls, who underwent an endoscopic biopsy, the median area under the receiver operating characteristic curve (AUROC) varied between 0.6 and 0.76 on validation data. Chierici et al. (7) trained ResNet neural networks on endoscopic images to identify pathological samples. The authors reported sensitivity and specificity above 0.9, when detecting pathological images vs. control. Stidham et al. (8) proposed and trained an inception neural network to assess UC disease severity from endoscopic images. The model assessment was compared against Mayo Scores assigned by human experts, and achieved 0.83 in sensitivity and 0.96 in specificity when classifying between remission and moderate-to-severe disease states. However, the aforementioned works required expensive and invasive techniques to collect patient data, thus limiting their clinical applicability.

Among the various techniques available to inspect the abdomen, auscultation, i.e., listening to body sounds with a stethoscope, is recognised as a particularly inexpensive and non-invasive approach. Manual auscultation of bowel sounds (BS) using a stethoscope was introduced by Laënnec in the 18th century and is today a standard clinical practice, performed during preliminary examinations (9, 10). BS are characterised by a short-time sound event in the range of 18 ms–3 s (11, 12). Reduced amount of BS events could indicate late states of bowel obstruction or paralytic ileus, while IBD or gastroenteritis could result in more frequent BS occurrences. However, clinical assessments based on BS remain difficult to date, due to the rather qualitative, manual evaluation of BS and a lack of quantification of BS acoustic properties (13). Especially for IBD, there are currently no data or literature studies that describe BS patterns for individual IBD stages. Similarly, IBD evaluation based on manual auscultation is not yet included in official recommendations for IBD diagnosis and monitoring (3). Previous investigations showed already that BS characteristics could support the diagnosis of GI disorders, e.g., by detecting abnormal BS patterns (14). For example, Craine et al. (15) collected and compared BS from patients with irritable bowel syndrome (IBS) and healthy controls. Their statistical analysis showed that the time interval between consecutive BS was significantly different between study groups with emptied stomach (fasting phase). Consequently, the authors proposed a threshold-based algorithm to detect IBS. In a later study (16), the authors included BS recorded from patients with CD and suggested that further BS time interval ranges should be used to discriminate IBS and CD. However, their analysis was conducted on short, 2 min audio recordings.

Since manual auscultation is usually performed for a few minutes only, limited diagnostic information of gastrointestinal conditions is retrieved, as reported by Ranta et al. (17). Due to irregular BS occurrences and varying abdominal location, the authors suggested to extend the BS observation period to at least 1 h. Du et al. (11) recorded 160 min of audio data and evaluated IBS classification in 15 patients with IBS and 15 healthy participants. BS were identified by applying an energy threshold to different signal frequency bands (18). The results showed 0.90 sensitivity and 0.92 specificity in leave-one-out cross-validation (CV) experiments. However, the authors did not address methods to deal with noise artefacts, which could render both, BS detection and classification, sensitive to errors during continuous abdominal recordings. Moreover, Du et al. verified BS detection in a small data fraction of 18 BS and 8 environmental sounds only. Spiegel et al. (19) designed a disposable wearable microphone to record BS from patients, who tolerated feeding after surgical intervention, patients with absent bowel function, i.e., postoperative ileus (POI), and healthy controls. The analysis of BS patterns showed that POI and non-POI patient groups were statistically different. Yao and Tai (20) compared BS characteristics collected from 5 min recordings across 16 patients with CD, 22 patients with UC, and 20 healthy controls. The authors reported that BS peak frequency and sound index, i.e., amount of BS events per unit time, could be correlated with disease activity. Although the studies mentioned above showed that BS information could be exploited to predict GI disorders, none of the works proposed an automated method for IBD vs. normal GI condition classification using acoustic features of BS events.

In this work, we investigated how naturally rare BS event spotting can be applied to derive acoustic features for IBD classification in continuous abdominal audio recordings. Our evaluation included 24 patients with IBD with varying disease activity and 21 healthy participants with no GI disorders. BS events were annotated in the audio recordings by pairs of expert raters. The annotations were used to train a deep-learning pattern spotting algorithm to detect BS events. Subsequently, we derived acoustic features from the detected BS events and trained a Gradient Boosting Classifier (GBC) to classify patients with IBD vs. healthy controls. We analysed the minimum audio recording duration required by our model to accurately detect IBD, as well as feature relevance and the link to IBD activity.

## 2 Materials and methods

**Figure 1:**
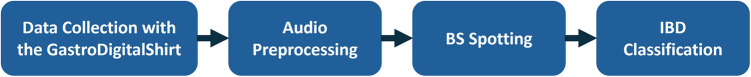
Method overview. BS were recorded with the GastroDigitalShirt for 1 h before and 1 h after breakfast. The audio recordings were preprocessed and split into 10 s audio segments. A BS spotting model marked BS events with a temporal resolution of 10 ms. Acoustic features were extracted from the BS events to classify patients with IBD from healthy participants.

Figure 1 provides an overview on the IBS classification. In the following subsections, we detail the data analysis, the processing steps, and their implementation.

### 2.1 Study procedure

Healthy participants and patients with IBD were recruited within a clinical study approved by the Ethics Commission of the Friedrich-Alexander Universität Erlangen-Nürnberg. To take part in the study, participants had to be at least 18 years old and tolerate the meals served during the study. Pregnant or breastfeeding individuals and patients with UC that underwent a total colectomy were excluded from the study. Our evaluation study included 21 healthy participants and 24 patients with IBD, recruited between March 2020 and November 2021. Among the patients, 14 were diagnosed with CD and 10 with UC. For each patient, faecal calprotectin (fCP), C-reactive protein (CRP) concentrations, and leukocyte counts were analysed from blood and stool samples to assess IBD activity. Disease activity was assessed based on fCP concentration. If the stool marker concentration was above 250 μg/g, the patient was considered to have active inflammation. Otherwise, the patient was considered in remission. Based on biomarker levels, 14 patients showed disease activity (see Supplementary Section S3.2 for more details). At the time of recording, three patients reported abdominal pain, one patient had joint and muscular pain in the lower abdomen region, one patient reported bloating, and one patient had pain while breathing. Table 1 illustrates the population characteristics of our dataset. Further details about IBD characteristics in the patient cohort can be found in Supplementary Table S2.

**Table 1:** Characteristics of the population included in this study.

Cohort	IBD	UC	CD	IBD activity	IBD remission	Healthy
Participants (n)	24	10	14	14	10	21
Annotated (n)	9	6	3	6	3	18
Sex, (%)						
Male	11 (45.8%)	3 (30.0%)	8 (57.1%)	7 (50.0%)	4 (40.0%)	13 (61.9%)
Female	13 (54.2%)	7 (70.0%)	6 (42.9%)	7 (50.0%)	6 (60.0%)	8 (38.1%)
Age [median years (range)]	39 (19–69)	38.5 (22–69)	39 (19–67)	33 (19–69)	46 (26–67)	28 (21–72)
BMI [median ${\mathrm{kg}/m}^{2}$ (range)]	23.8 (17.4–32.1)	22.2 (17.9–26.0)	24.5 (17.4–32.1)	23.8 (17.4–32.1)	24.0 (18.4–32.1)	22.5 (17.2–32.2)

BMI, body mass index.

Study sessions began in the morning before breakfast in the lab at approximately 7:30, after participants provided written consent. BS were recorded continuously for 1 h before (fasting phase) and 1 h after breakfast (postprandial phase), including meal intake. Participants could interrupt the recordings anytime, e.g., for toilet visits. The GastroDigitalShirt (21), a smart T-shirt embedding eight miniaturised microphones (Knowles, SPH0645LM4H-B) connected to a belt-worn computer (Radxa, Rock Pi S), was used to collect BS at sampling frequency $f_{s}=16$ kHz. The integrated microphone array was positioned according to the nine-quadrant abdominal maps (see Supplementary Figure S1, Section S2 for details). Different shirt sizes were provided to fit all participants, and stretchable fabric based on elastane was used to ensure optimal comfort and sensor–skin interface. During the recording, participants were asked to relax on a lounger, while reading, using audio or video entertainment on a tablet, or sleeping. To avoid peristalsis overstimulation, participants could not drink caffeine-based beverages, e.g., coffee or tea, and laid down for most of the recording. Nevertheless, the participants sat at a table to eat breakfast and could often have conversations with the study personnel. In addition, drinking water was allowed throughout the whole session. Besides audio during talking and eating, motion sounds and different environmental sounds were captured, including traffic and voices outside the recording room.

### 2.2 BS annotation

Pairs of expert raters reviewed and annotated BS events in recordings of a data subset, including 27 participants (18 healthy, 9 patients with IBD, of which 3 patients were in biochemical remission). BS were marked in the audio data by visually and acoustically inspecting the signal using the software Audacity. Empirically, microphones placed on the stomach and small intestine collected most of BS events and showed the largest signal-to-noise ratio (SNR). Therefore, the microphone positions at the stomach and small intestine were included in the annotation. Since IBD, especially UC, are often located between small and large intestines, an additional microphone placed at the distal part of the large intestine (CH7 according to Supplementary Figure S1) was annotated for patients with IBD and a subset of the healthy group. The channel at the large intestine could not be annotated for 10 healthy participants due to SNR limitations. Every channel was labelled separately, since BS could occur at one or more channels depending on sound propagation in the abdomen (22). Based on the literature-reported temporal features (12, 23) as well as preliminary annotation reviews, raters discussed and agreed on the BS labelling approach: BS duration must be ≥18 ms, and consecutive BS with sound-to-sound interval <100 ms were labelled as one event. BS with noisy temporal patterns were labelled as tentative and were excluded from the analysis.

Depending on BS temporal occurrence, 1 h of audio could require 8–12 h per rater to label all BS events. Therefore, audio was annotated by one of the raters and labels were reviewed by another one. A subset comprising the first 30 min of audio from eight healthy participants and nine patients was used to evaluate annotation quality. Inter-rater agreement, i.e., Cohen’s κ score, was used to analyse agreement on BS labels. Cohen’s κ was computed on the subset with a time resolution of $\frac{1}{f_{s}}\approx$ 0.06 ms. The evaluation yielded a substantial rater agreement, with Cohen’s κ score of 0.70 for the healthy group and 0.73 for the patient group. On approximately 136 h of audio, 11,482 BS were annotated by expert raters.

### 2.3 BS spotting

**Figure 2:**
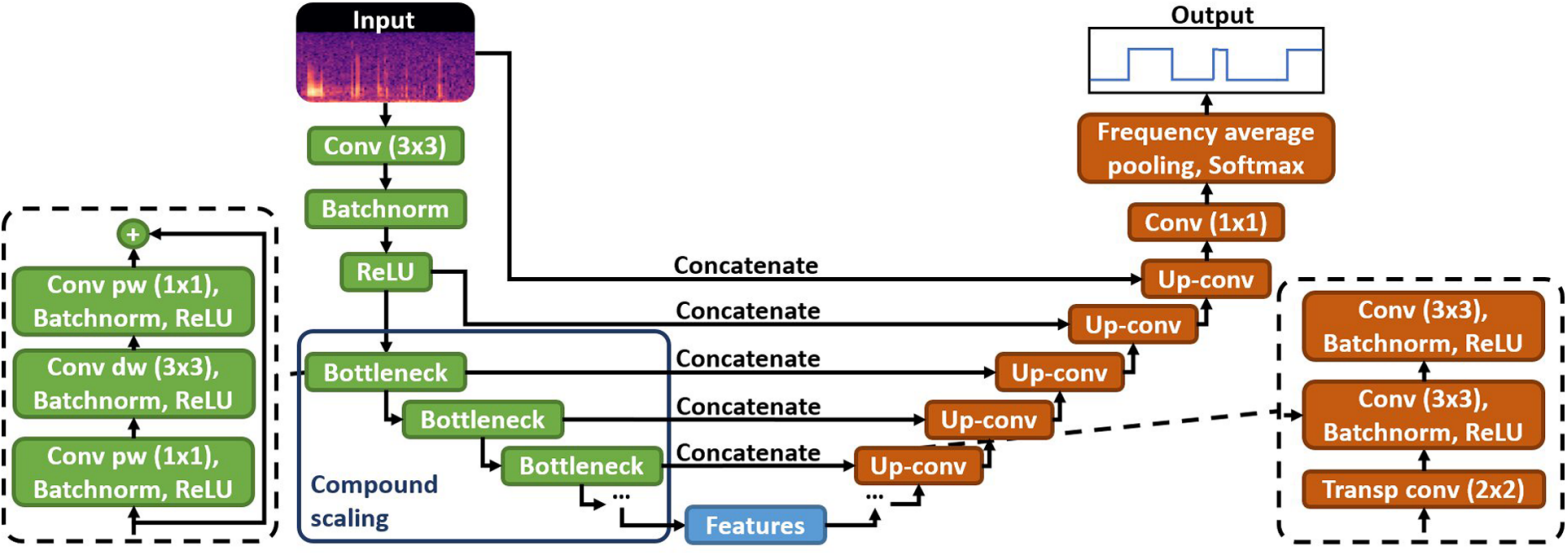
Architecture of the EffUNet model for BS spotting. EffUNet input are audio spectrograms, outputs are temporal binary masks labelling each spectrogram frame as containing either BS or not. The EfficientNet-B2 encoder (green boxes) extracts relevant acoustic features that are subsequently upsampled and located on the spectrogram by the decoder (orange boxes) to identify BS occurrences on the time axis. Conv, convolution; dw, depthwise; pw, pointwise; batchnorm, batch normalisation; transp, transposed.

Expert annotations were used to train an Efficient-U-Net (EffUNet) model for BS event spotting (24). The neural network architecture was adapted from the UNet model (25), originally designed for biomedical image segmentation. UNet models are composed by an encoder and a decoder. The encoder extracts relevant features from the input data and the decoder helps locating them on the original data by applying consecutive upsampling and concatenation operations (see Figure 2). We selected EfficientNet-B2 (26) as the encoder for our UNet model (hence the name EffUNet) as it showed promising results in spotting BS in continuous recordings (24). EffUNet took as an input an audio clip of 10 s converted to log Mel-spectrogram representation and returned a binary time mask marking each spectrogram frame as either containing BS or not. Figure 2 illustrates EffUNet architecture.

Recordings were preprocessed by applying a high-pass biquadratic filter (cutoff: 60 Hz) to remove offsets, and split into non-overlapping audio segments of 10 s. Audio segments were subsequently converted to log Mel-spectrograms using a 25 ms sliding window with 10 ms hop size (preprocessing: Hanning windowing) and 128 Mel-bins. Consequently, temporal resolution of the EffUNet-retrieved BS was 25 ms. The obtained audio spectrograms were 0-padded along the time dimension to 1,056 frames to match the required input size of EffUNet. A spotted BS event was derived as a set of consecutive overlapping spectrogram frames that were detected by EffUNet as containing BS.

Transfer learning was applied to EffUNet encoder, i.e., we initialised the encoder weights with EfficientNet-B2 pretrained on AudioSet dataset (27, 28), comprising more than 500 different sounds (including BS) and over 5,000 h of audio split into 10 s audio clips sampled at 16 kHz. Since AudioSet only provides weak audio labels, no pretraining could be applied to the EffUNet decoder, therefore He initialisation (29) was used. Our previous experiments showed that transfer learning can improve BS spotting performance (30).

Leave-One-Participant-Out (LOPO) CV was used to train EffUNet on the expert-annotated data subset. Following the Pretraining, Sampling, Labeling, and Aggregation (PSLA) pipeline (27), EffUNet was trained for 25 epochs with an imbalanced batch size of 32. The initial learning rate of $1\times 10^{-4}$ was subsequently reduced from the sixth epoch with a decay of 0.85 at each epoch. We used Adam optimiser (31) with $\beta_{1}=0.95$, $\beta_{2}=0.999$, and weight decay of $5\times 10^{-7}$. The sum of cross-entropy loss and dice loss was used as the loss function during the optimisation. The model obtained at the end of the training was used to detect BS on the held-out participant recording. The inference was run on the three channels covered by the annotation, regardless of whether or not BS were labelled for the third sensor on the large intestine (CH7). Thus, the same audio channels were used for the BS spotting inference of patients and healthy controls. To spot BS on the unlabelled audio data of the full dataset, EffUNet was retrained on the entire expert-annotated data subset. BS events were retrieved for the same channels used for annotation.

Separate data augmentation operations were applied to the samples of each training batch. Time-frequency masking (32) was randomly applied to a maximum of 24 frequency bins and a maximum of 10% of the spectrogram frames. White noise was randomly added to the input. Spectrogram frames could be shifted by up to ±10 time bins.

Spotting performance for the LOPO CV was derived across all annotated data with 72% precision and 73% recall. Further details of the BS spotting method and performance evaluation can be found in Baronetto et al. (24).

**Figure 3:**
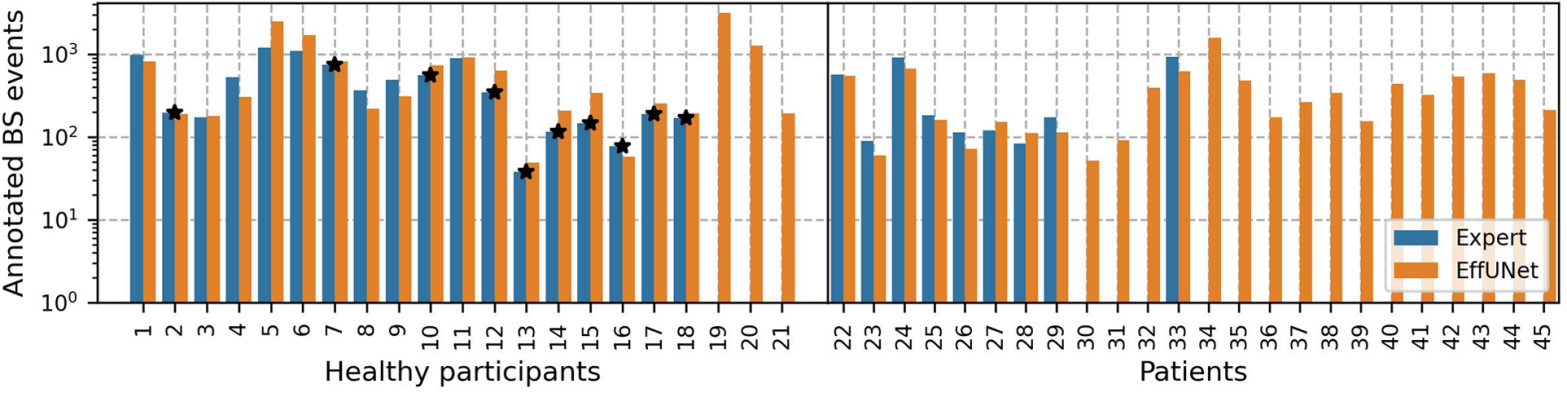
Annotated BS events per participant. Pairs of raters annotated recordings in a sub-dataset, including 27 participants, in total 11,482 BS events. Our spotting model EffUNet detected 23,650 BS on the full dataset, comprising 45 participants. Participants whose channel on the large intestine could not be manually annotated are marked by a star.

The full dataset comprising 45 participants was subsequently processed with our spotting model to detect BS. On approx. 281 h of audio, EffUNet identified 23,650 BS events. Figure 3 shows the per participant BS events annotated by expert raters and those spotted by EffUNet. For the annotated data subset, expert raters and EffUNet spotting identified a similar amount of BS.

### 2.4 Audio preprocessing and BS feature extraction

Audio was preprocessed using a 0-phase Butterworth band-pass filter of eighth order (passband frequency: 60–5,000 Hz) following previous works on BS analysis (12, 23). BS events were extracted from the audio recording according to expert annotations and EffUNet spotting results. BS event duration was padded with a surrounding audio signal to fit the frame length for feature calculation. A sliding window of 32 ms length and a hop size of 8 ms was used to calculate temporal, spectral, and perceptual features. The features obtained from each BS event across the sliding windows were subsequently averaged. A detailed list of the features can be found in Supplementary Section S2.

Recordings were split into non-overlapping classification windows $S_{i}$ of duration δ = 10 min. BS events within the same classification window $S_{i}$ were grouped and mean and variance statistics were calculated for every BS feature. Classification windows that did not include BS events were omitted. For the GBC training, only selected features based on feature mutual information (33) were used.

The retained classification windows $S_{i}$ were subsequently labelled as “healthy” if they were collected from the healthy population, otherwise they were marked as “IBD.” Standardisation was applied to the features before the GBC training. In total, we obtained 704 classification windows (234 $S_{i}$ for patient group) on the expert-annotated data subset and 1,344 classification windows (682 $S_{i}$ for patient group) across the full dataset.

Furthermore, we investigated the amount of audio data needed to classify patients with IBD vs. healthy controls by varying the classification window $S_{i}$ duration δ between 1 s and 10 min. For each duration δ, GBC was retrained and evaluated using the selected BS features.

### 2.5 IBD classification

We developed a binary GBC (34) to identify classification windows $S_{i}$ recorded from patients with IBD vs. healthy controls. On the annotated data subset, the model was trained using 50 estimators, exponential loss, and a learning rate of $1\times 10^{-4}$, i.e., weighting factor applied to the new trees created during model training. When training on the full dataset, we increased GBC estimators to 100 and the learning rate to 0.001. During the training, the Friedman mean squared error score was employed as the criterion to evaluate the quality of a split. The classifier was evaluated on the dataset using a stratified group k CV, where k was chosen based on the amount of participants included in the BS annotation. To balance the two classes, Synthetic Minority Oversampling TEchnique (SMOTE) resampling (35) was applied to the trainset of each fold during training.

The classified windows $S_{i}$ were merged for every participant using hard majority voting. Hence, a participant was classified as a patient with IBD if at least 50% of the classification windows were detected as being recorded from a patient with IBD. The participant was classified as healthy otherwise.

### 2.6 Evaluation metrics

IBD classifier performance was evaluated across all k CV folds by computing the AUROC, sensitivity, and specificity.

The IBD classification score was subsequently compared with the stool and blood inflammation biomarkers to analyse the correlation between the IBD class probability of the GBC model and disease activity across all patients. Spearman’s r correlation coefficient was used to investigate the relationship between our model IBD score and biomarker levels. The IBD classification score was obtained by averaging the IBD class probability across all classification windows $S_{i}$ for every patient.

Mean and standard deviation (SD) of AUROC were analysed to find the optimal classification window duration δ, i.e., minimum amount of audio to analyse to detect IBD, yielding the largest mean AUROC and smallest standard deviation of AUROC across all experiments.

## 3 Results

### 3.1 BS feature selection

**Figure 4:**
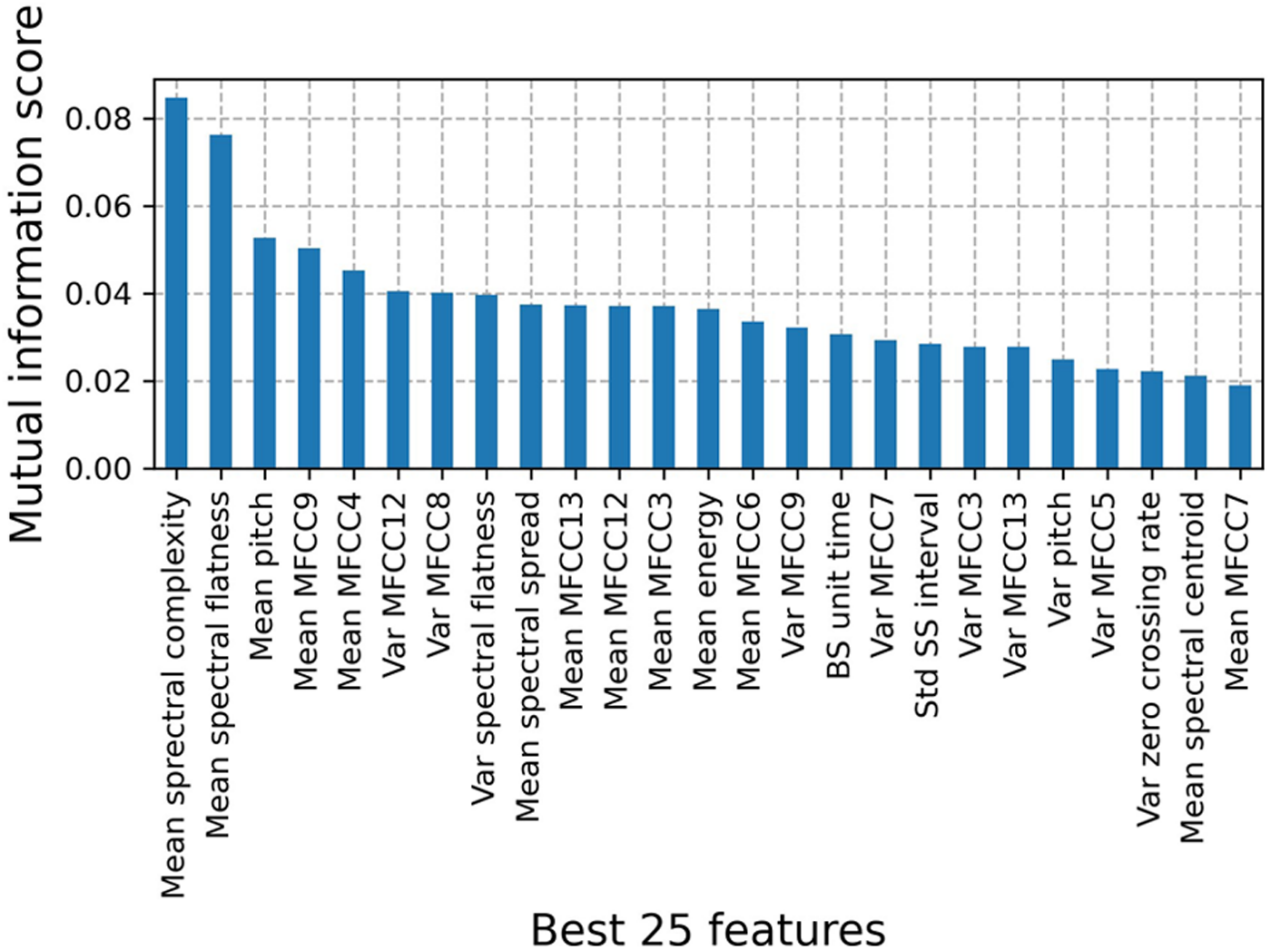
Mutual information scores for the top 25 features to classify patients with IBD vs. healthy controls on the full dataset. Features were derived for classification windows $S_{i}$ of duration δ = 10 min that contain BS events. Among the best features, we manually selected 11 MFCCs to train the IBD classification model.

Figure 4 shows the top 25 BS features to classify patients with IBD vs. healthy controls on the full dataset. Based on the mutual information score, we selected the mean of 11 Mel Frequency Cepstral Coefficients (MFCCs) to subsequently train a GBC. The first two MFCCs were excluded from the classification as BS information contained in those frequency bins was filtered out during the preprocessing. Feature definitions and mutual information scores for the annotated subset can be found in Supplementary Section S2.

### 3.2 IBD classification

**Figure 5:**
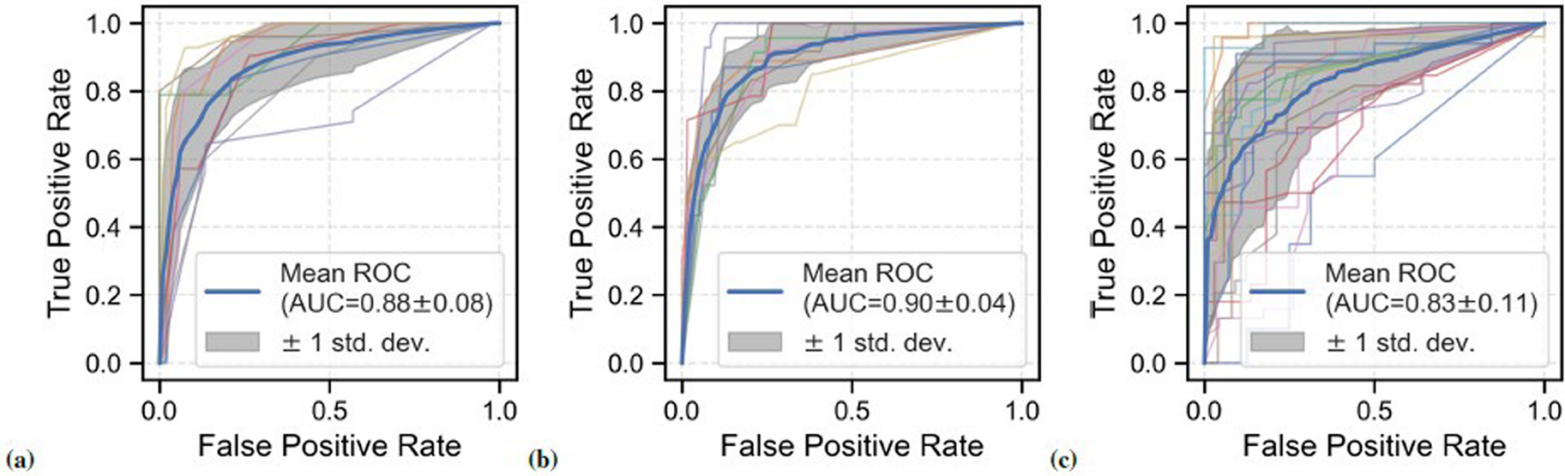
ROC curves for the BS-based classification of patients with IBD vs. healthy controls across all CV folds. Different data configurations were investigated. (**A**) Performance on data subset based on expert BS annotations (27 participants, 9 patients with IBD), mean AUROC: 0.88. (**B**) Performance on the annotated data subset using EffUNet BS event spotting (27 participants, 9 patients with IBD), mean AUROC: 0.90. (**C**) Performance on full dataset using EffUNet BS event spotting (21 healthy controls and 24 patients with IBD), mean AUROC: 0.83.

IBD classification performance on the annotated data subset of 27 participants is shown in Figures 5A,B. Stratified group ninefold CV was used for evaluation, i.e., each CV fold contained classification windows $S_{i}$ from one patient only. For the expert-annotated sub-dataset, GBC yielded a mean AUROC of 0.88 (SD = 0.08), mean sensitivity of 0.81 (SD = 0.12), and mean specificity of 0.88 (SD = 0.09) across all CV experiments. A similar performance was observed for BS events detected by EffUNet on the annotated data subset: mean AUROC: 0.90 (SD = 0.04), and mean sensitivity: 0.88 (SD = 0.13), mean specificity: 0.84 (SD = 0.08).

Figure 5C shows the classification performance on the full dataset with BS events spotted by EffUNet. The model was trained and tested using a group stratified 21-fold, i.e., each validation fold included data of one patient and at least one healthy participant. A mean AUROC of 0.83 (SD = 0.11) was reached across all CV experiments, with mean sensitivity of 0.80 (SD = 0.15) and mean specificity of 0.83 (SD = 0.13). Compared to the expert-annotated sub-dataset, the AUROC SD was larger on the full dataset. Comparably low performance was observed for two patients with CD with AUROC of 0.55 and 0.65, respectively.

We further benchmarked our GBC model using noisy audio data. Moreover, we analysed the class separability between CD and UC by retraining our GBC classified on the patient group only. The results of our experiments can be found in Supplementary Section S3.4.

### 3.3 IBD classification with majority voting

Majority voting yielded an accuracy of 0.93 for the data subset using expert annotations, with a sensitivity of 0.89 and a specificity of 0.94. When using EffUNet to spot BS events on the annotated data subset, accuracy, sensitivity, and specificity reached maximum scores. For the full dataset EffUNet BS event spotting yielded an accuracy of 0.93, sensitivity of 0.88, and specificity of 1.

### 3.4 Minimum data to detect IBD

**Figure 6:**
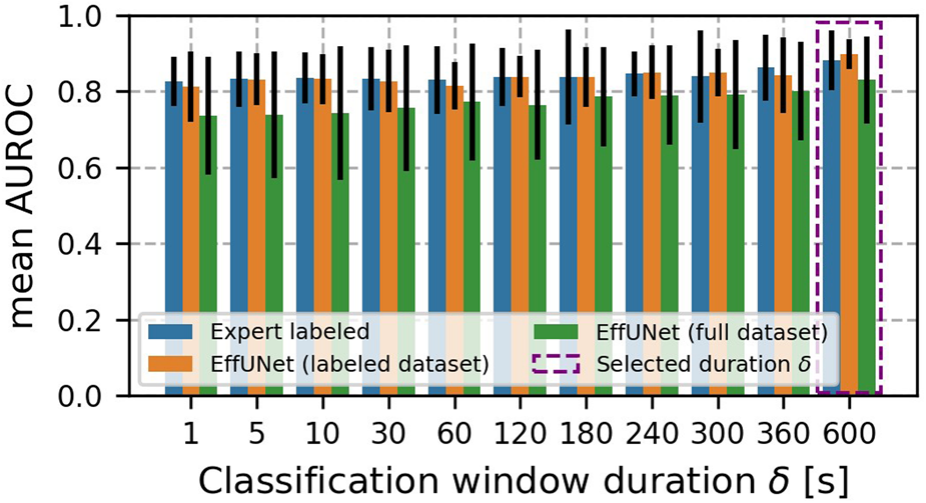
Mean AUROC across classification window duration δ. Error bars indicate AUROC SD. For all data configurations, mean AUROC increased with classification windows duration δ. The best mean AUROC was found for δ = 10 min. Along with the δ increase, the AUROC SD decreased and was minimum for δ = 10 min.

Figure 6 shows the mean AUROC scores. For all data configurations, mean AUROC increased with the duration of classification windows. The best mean AUROC was found for δ = 10 min. Along with the δ increase, the AUROC SD decreased and was minimum for δ = 10 min.

### 3.5 Further analysis

For the IBD classification based on the expert-annotated data subset the correlation with leukocyte counts was moderate (r = 0.47). The analysis is further detailed in Supplementary Section S3.

## 4 Discussion

Current clinical diagnosis and monitoring of IBD relies on a combination of clinical, imaging, and biochemical assessments. Due to the variety of investigations needed and, consequently, the time and effort required, IBD diagnosis may be affected by delays (36). Our work aims to demonstrate the relevance of BS event spotting. Here in particular, we demonstrate how to differentiate IBD from healthy GI conditions non-invasively, using BS events extracted from continuous abdominal audio recordings. We investigated acoustic features and a GBC model to classify short, 10 min audio recordings in patients with IBD vs. healthy controls. While the recording shirts for the present work were custom-made, the wearable monitoring device could be inconspicuous, low-cost, and operate continuously, at least for 10 min episodes, to collect BS across different digestive phases. Our approach is inexpensive and can monitor GI processes continuously, thus avoiding repeated and uncomfortable abdominal assessments. We believe that our approach has the potential to be employed as a screening test and telemedicine solution for bowel disorders.

Our analysis of acoustic BS features showed that IBD can be detected with a mean sensitivity of 0.81 and a mean specificity of 0.88 using expert annotations (see Figure 5A). When spotting BS events with EffUNet on the annotated data subset, performance was almost perfect, hence reproducing the expert annotations. For the full dataset, GBC performance decreased slightly in AUROC, from 0.88 for the annotated data subset to 0.83. A performance decrease for the full dataset of 5% was expected due to a potential higher amount of false positives retrieved as BS events. Given the limited annotation on the full dataset, not all participant recordings could be used for EffUNet training. Consequently, EffUNet retrieval performance, i.e., false positive rate, could not be evaluated on the full dataset. While in this study expert annotations were employed to train our IBD classifier, the classification results obtained with EffUNet-retrieved events show that our method could be fully automated without requiring manual expert input, rendering its clinical deployment feasible.

By-participant AUROC results showed that some patients were harder to classify correctly than others. In particular, AUROC dropped to 0.65 and 0.55 for two patients with CD (IDs: 40 and 44). One patient (ID: 40) was clinically obese (BMI: 31.2 ${\mathrm{kg}/m}^{2}$). Previous studies with obese patients (37) reported difficulty in abdominal auscultation due to the thick adipose tissue layer, which could have similarly affected our wearable BS recording. A drop in BS spotting performance was reported in recent studies involving obese patients too, e.g., Zhao et al. (38). Although no additional endoscopic assessment was performed at the time of study recruitment, the patient assessments based on questionnaires and inflammation biomarker levels indicated disease remission. In addition, the clinically obese patient in our study (ID: 40) had previously undergone an ileocaecal resection, which may have affected inflammation and acoustic recordings. Patients with UC, who previously received a total colectomy, were not included in the study as no further intestinal inflammation is to be expected. The second patient with CD (ID: 44) with AUROC below 0.70 was underweight (BMI: 17.4 ${\mathrm{kg}/m}^{2}$). Although the patient’s biochemical assessment showed active inflammation, the low IBD class probability (mean class probability was approximately 0.50) could have been caused by the recording setting. While different GastroDigitalShirt sizes were available for the study, even the smallest size may have insufficiently fitted the patient, thus potentially decreasing captured abnormal BS events.

We evaluated our IBD detection method for its potential as a screening test and thus analysed the classification performance on the study population besides the classification window dataset. To obtain one IBD classification per participant, we merged classification window results for each participant with majority voting. Regardless of whether BS were retrieved manually or using EffUNet, IBD detection yielded an accuracy ≥0.93. However, overall five patients were not detected with the majority voting strategy, resulting in a sensitivity of 0.89 on the annotated data subset and 0.88 on the full dataset. Future work may explore alternative postprocessing techniques. For instance, soft majority voting could be used instead of the hard voting applied in the present work.

In standard manual auscultation, it is recommended listening to BS for a few minutes (10). However, when no BS can be heard, auscultation should be prolonged up to ≈ 10 min, to maximise chances to hear BS events (13). Our performance analysis over the classification window duration δ confirms common clinical practice, as the highest mean AUROC and lowest SD AUROC was obtained for δ = 10 min (see Figure 6). Although the classification window duration <1 min yielded mean AUROC above 0.74 regardless of the BS retrieval method used, GBC performance was more variable, especially when EffUNet annotations were employed. As BS could occur sparsely over time, using classification window duration δ of a few seconds up to 5 min may capture a few BS events only or even none at all. Consequently, the statistics obtained from the extracted features could be insufficient to detect IBD. Our results agree with past investigations on BS feature analysis (17), where it was recommended to analyse BS according to an hour-long recording protocol to maximise collected BS amount. Nevertheless, we believe that δ = 10 min is a reasonable window choice that maximises chances for a low-artefact recording, e.g., during sedentary moments with low motion and acoustic noise level.

To minimise the invasive character of clinical IBD monitoring, biomarkers from blood and stool samples are often used (39). We jointly analysed the per-patient mean IBD class probability and corresponding biomarker levels to understand potential relations (see Supplementary Section S3.2 for more details). Except for leukocyte counts, where correlation was moderate for the annotated data subset, no correlation was found. Our patient cohort comprised those with active inflammation and those in remission, based on biomarker levels (see Supplementary Section S3.2). To achieve robust classification performance, patients with any inflammation state were included in GBC model training, thus maximally utilising the available data. Therefore, we hypothesise that our IBD classifier might be unable to distinguish between different inflammation states. Furthermore, recent studies (40) reported that up to 60% of patients with IBD in remission may still experience symptoms. Our analysis of biomarker levels among misclassified patients revealed no difference in classification performance between patients with active disease and patients in remission (see Supplementary Figure S3). Thus, our IBD classifier could identify patients with IBD vs. healthy controls regardless of the disease activity.

We further investigated whether our BS event analysis method could be used to robustly discriminate between patients with CD and UC (see Supplementary Section S3.3). However, our approach yielded a model biased towards the CD class and failed to perform, which could be due to insufficient number of patients in the study, issues in patient characterisation, or inadequate BS features, which were intended for the IBD vs. healthy classification. IBD type diagnosis is still challenging, even with traditional assessments, e.g., for indeterminate colitis (41). Future studies should expand the BS feature analysis across larger IBD populations as well as other biomarker types (39, 42), including various disease activity states.

We tested the trained GBC models on noise data segments of the patients’ recordings to confirm that the training did not capture audio properties other than those of the BS patterns. The AUROC results suggest that the GBC models were unable to classify the noise segments into patient vs. healthy categories. Thus, our noise test indicates that the GBC could detect IBD based on relevant audio information derived from BS features. See Supplementary Section S3.4 for details.

To treat IBD and monitor the disease activity, it is important to identify individual inflammation regions. Thus, it would be beneficial to locate BS sources while diagnosing IBD. However, previous studies on abdominal sound propagation showed that estimating BS source location is challenging, even when employing multiple sensor recordings (22). Dimoulas (43) proposed a 2D source localisation approach based on piezoelectric and inertial sensors, but did not validate the approach *in vivo*. Our preliminary annotations confirmed that not all sensors could capture the same events due to sound absorption within the abdomen. Therefore, each channel was annotated separately. Because of the time-consuming process in manually labelling single BS events, only those channels with the most BS events could be included in our analysis. However, analysing BS at different abdominal locations could improve the IBD detection performance, as abnormal BS patterns originated by the inflamed region may be more representative. Further investigations are necessary to successfully map BS source on the abdomen. For instance, BS recording could be combined with imaging, techniques to establish source location ground truth, e.g., as seen in Saito et al. (44), and develop source localisation methods leveraging microphone arrays.

In this work, we designed and evaluated a method to distinguish IBD from healthy condition based on BS event spotting. For the first time, we demonstrated that information derived from BS events could be potentially applied to a clinical condition. Our results warrant further investigations to better describe BS event properties with respect to the varying inflammatory bowel conditions. Since no patients with other GI disorders, e.g., IBS or gastroenteritis, were recruited within the clinical study, we did not evaluate the specificity of our method for IBD in comparison to other diseases. Based on the similar classification results of Du et al. (11) for IBS, we assume that our BS event spotting and feature extraction approach can be extended for IBS, too. Moreover, the relationship between BS characteristics and symptoms related to general abdominal discomfort was not analysed in this study, as only a small subset of patients reported abdominal pain at the time of the recording. Future work should include patients with various digestive disorders to test IBD classification performance vs. other diseases.

Due to SNR limitations, the sensor at the large intestine could not be manually annotated for all healthy controls. Additionally, the number of healthy individuals with manually annotated recordings was twice that of patients with IBD. Consequently, there was a data imbalance across individuals when training the GBC on the annotated data subset. However, we could retrieve BS events from the missing channel and the rest of the study population with EffUNet (see Section 2.3 for more details). To minimise data imbalance, the GBC training dataset was resampled using SMOTE. The AUROC analysis across the CV folds (see Figures 5A,B) showed that GBC achieved comparable IBD classification performance on the annotated data subset and full dataset (AUROC 0.88 vs. 0.83, respectively). Therefore, our results confirmed that data imbalance did not impact IBD detection, but also that an automated spotting method for BS event retrieval and analysis could overcome challenges in the manual examination of audio data. Data imbalance is to be expected in real-world scenarios, considering the IBD prevalence in the adult population (2).

Our work presents a prospective non-invasive, continuous test for potential patients with IBD based on the analysis of acoustic BS features. We collected BS across patients with IBD with different disease conditions as well as healthy controls. We selected most relevant features and trained a GBC to detect audio classification windows of 10 min collected from IBD vs. healthy controls. Our method could detect patients with IBD with mean AUROC above 0.83 regardless of the annotation tool employed to mark BS in the audio recordings. Our analysis of correlation between IBD probability and inflammation biomarker levels showed that there was a weak to moderate correlation depending on the biomarker and BS annotation tool. Our method was able to detect IBD with precision ≥ 0.93, demonstrating that it could be used in clinical practice as an IBD test.

## Data Availability

The datasets presented in this article are not readily available because the audio data includes voices and conversations during the recordings. Requests to access the datasets should be directed to Oliver Amft, oliver.amft@hahn-schickard.de.
