# Supplementary material for: Automated inflammatory bowel disease detection using wearable bowel sound event spotting
Source: Front Digit Health. 2025 Mar 13;7:1514757. doi: 10.3389/fdgth.2025.1514757 (PMC11965935; doi:10.3389/fdgth.2025.1514757)
Supplement: Supplementary file 1 [file Datasheet1.pdf]

Supplemental Material for Baronetto, Fischer et al., Automated Inflammatory Bowel Disease Detection Using Wearable Bowel Sound Event Spotting

1 GASTRODIGITALSHIRT SCHEMATICS

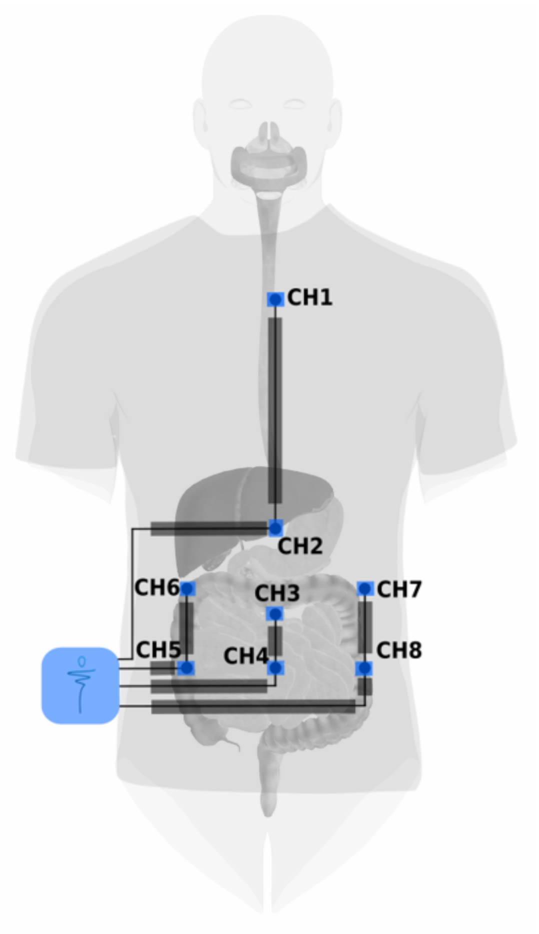

Figure S1. GastroDigitalShirt microphone matrix based on the 9-quadrants abdominal map reference (human model adapted from Genesis1 (2020), under Creative Commons Attribution 4.0 International License).

2 LIST OF BS FEATURES

Table S1 presents a detailed list of spectral, temporal and perceptual features we extracted from BS and used to detect IBD patients from on abdominal recordings. Within the analysis, common features employed

Table S1. BS features extracted investigated in this study. For each classification window, BS feature mean and, if applicable, variance (or std.) were calculated from the BS events within the window.

**Spectral Features**	
Mel Frequency Cepstral Coefficients (MFCCs)	Signal frequency bands representation according to the mel-scale, i.e. human auditory system. In this work, the first 13 coefficients were employed for the classification.
Spectral centroid	The center of mass of a signal spectrum. Perceptually, the spectral centroid is related to sound brightness.
Spectral complexity	A measurement of how much information is needed to estimate the signal spectrum, it is based on the amount of spectrum peaks.
Spectral flatness	How much a signal spectrum is noise-like, e.g. white noise has value 1, or similar to a pure tone sound, which has value 0.
Spectral spread	It defines the signal spectrum variance around the spectral centroid.
Strong peak ratio	The ratio between the maximum peak's magnitude and the peak bandwidth in a spectrum. The higher the ratio, the more pronounced is the peak. We estimated the ratio according to Gouyon and Herrera Gouyon and Herrera (2001).
Maximum magnitude frequency	It measures the frequency having the largest magnitude in a spectrum.
**Temporal Features**	
Signal energy	
Zero crossing rate	Number of time the signal change from positive to negative and vice-versa per unit time.
**Perceptual Features**	
Dissonance	Perceptual roughness of an audio signal based on the spectrum peaks. The dissonance was estimated according to Plomp and Levelt Plomp and Levelt (1965).
Inharmonicity	How much harmonic a signal is based on the signal fundamental frequency and other spectrum peaks.
Signal pitch	Signal fundamental frequency. The Yin algorithm De Cheveigné and Kawahara (2002) was used for the estimation.
**Other Features**	
BS location	It is defined as the location of the sensor that recorded the BS. Sensor location was described according to the GastroDigitalShirt sensor matrix, see Fig. S1.
BS per unit time	Number of BS events occurring within a classification window, i.e. portion of audio recording of duration δ.
Sound-to-Sound (SS) interval	Time interval in seconds occurring between consecutive BS events within a classification window.

for audio processing were tested. We additionally included other features, whose definition can be found in Tab. S1. Based on mutual information, the best features were selected to train the Gradient Boosting Classifier. Mutual information scores for the top 25 features to classify IBD patients vs. healthy controls for all data configurations are shown in Fig. S2.

3 FURTHER ANALYSIS

3.1 IBD Characteristics in Patient Cohort

Table S2 illustrates clinical characteristics of the patient population, including clinical scores based on reported patient symptoms and inflammation location in the GI tract.

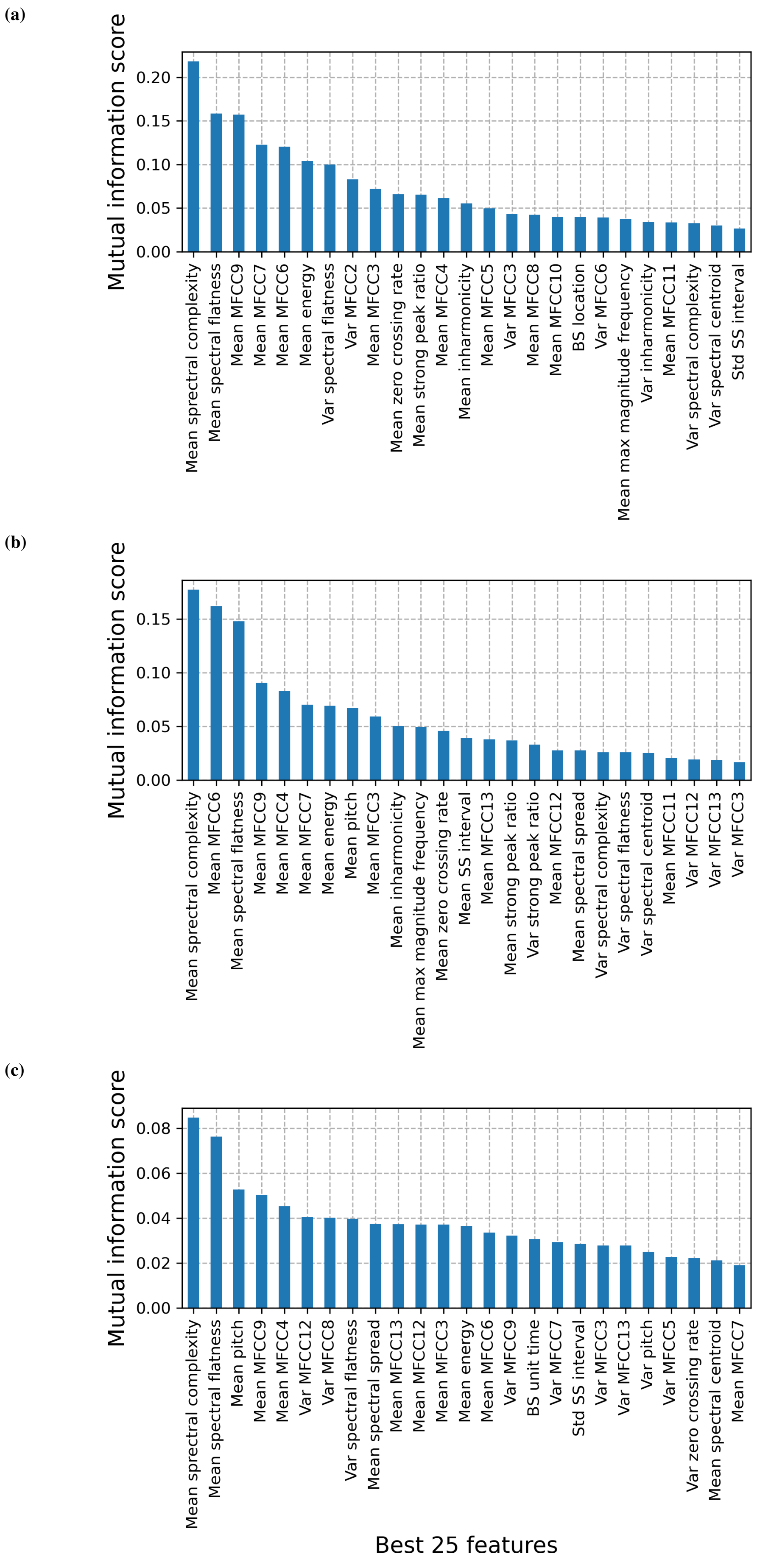

Figure S2. Mutual information scores for the top 25 features to classify IBD patients vs. healthy controls. Features were derived for classification windows S_i of duration δ=10 min that contain BS events. Among the best features, we manually selected 11 MFCCs to train the IBD classification model. (a) Expert annotated sub-dataset including 27 participants (9 IBD patients). (b) EffUNet-based detection of BS on the annotated data subset. (c) EffUNet-based detection on the full dataset (21 healthy participants and 24 IBD patients).

Table S2. Clinical characteristics of the IBD patient population included in this study. Med: median. IQR: inter-quartile range.

**Patient cohort**	**UC**	**CD**
6-point Mayo-Score, med (IQR)	4 (2.5)	N/A
partial Mayo-Score, med (IQR)	7 (2.75)	N/A
Montreal A, med (IQR)	N/A	2 (0)
Harvey-Bradshaw Index, med (IQR)	N/A	3.5 (3.8)
Location		
Terminal ileum, n	0	3
Ileocolon, n	0	7
Left colon (until colic flexure), n	5	0
Right transverse colon, n	0	1
Colon, n	0	3
Colon (pancolitis), n	3	0
Extended disease, n	1	0
Rectum, n	1	0

3.2 Correlation With Inflammation Biomarkers

Figure S3 shows the distribution of inflammation biomarkers collected from our IBD patient subset. Disease activity was assessed based on fCP concentration. If the stool marker concentration was above 250 μg/g, the patient was considered to have active inflammation. Otherwise, the disease was regarded as in remission.

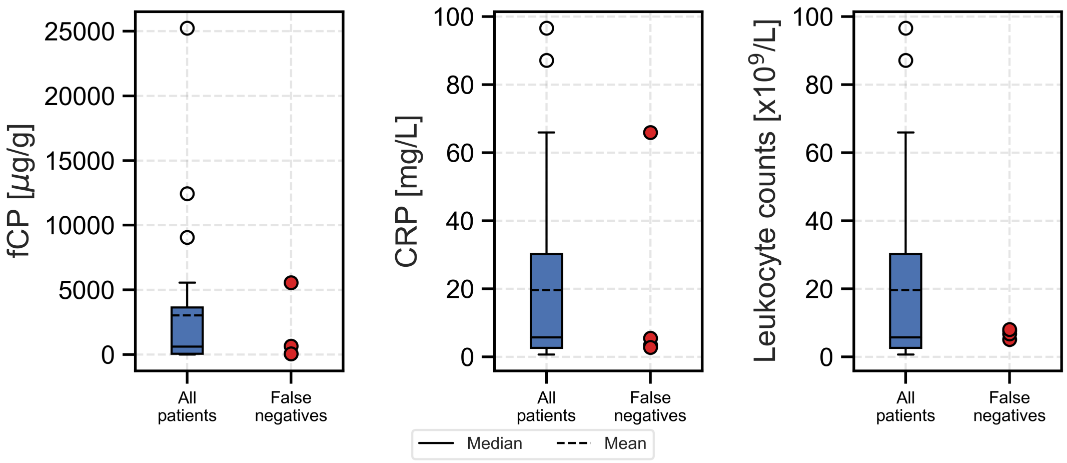

Figure S3. Inflammation biomarkers of all IBD patients and in relation to false negatives classification result across the full dataset.

To evaluate the correlation between our IBD classifier output and inflammation biomarkers, we obtained for each patient a classification score by averaging the IBD class probability across all classification windows S_i per individual. We analysed the correlation between classification class probability and inflammation biomarker levels using Spearman's r. For the IBD classification based on the expert annotated data subset, a very weak to no correlation was found with CRP (r=-0.12) and fCP (r=-0.02), while correlation leukocyte counts was moderate (r=0.47). For the annotated data subset using EffUNet BS event spotting (27-participants), a very weak correlation with CRP (r=-0.03) and with leukocyte counts (r=-0.10), and weak correlation with fCP (r=0.22) was found. Similarly, on the full dataset, correlation was very weak (r=-0.17 for CRP, r=0.02 for fCP, r=-0.01 for leukocyte counts).

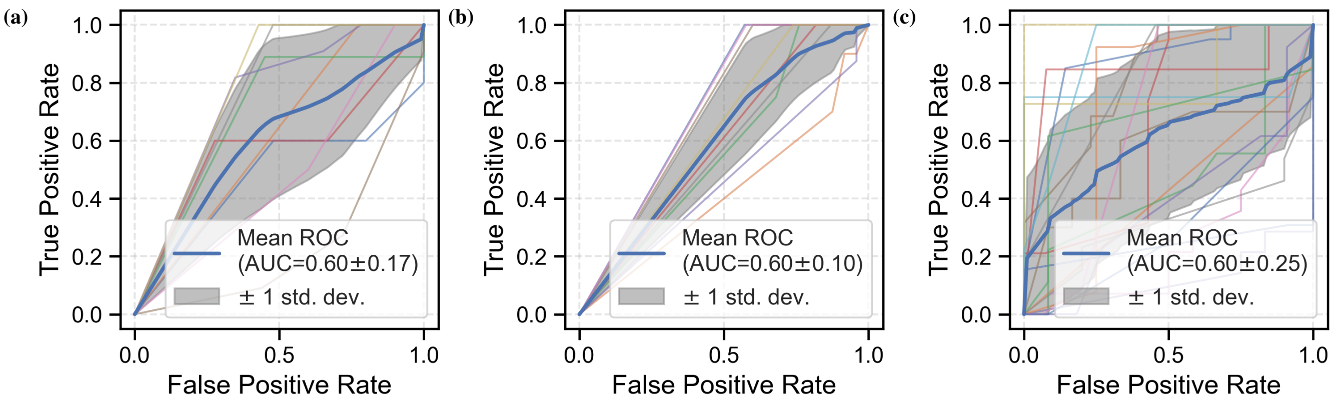

Figure S4. ROC curves for GBC test on noise data. To analyse if the classification model had learned unwanted non-BS sound patterns, the GBC model, as trained for IBD vs. healthy classification, was tested on non-BS audio segments across all CV folds. (a) Analysis for the annotated data subset with non-BS audio segments sampled excluding expert annotations (18 healthy participants and 9 IBD patients). (b) Analysis for the annotated data subset with non-BS audio segments sampled excluding EffUNet BS spotting results. (c) Analysis for the full dataset with non-BS audio segments sampled excluding EffUNet BS spotting results (21 healthy participants and 24 IBD patients).

IBD inflammation assessment based on biomarkers can be challenging, as biomarker concentration ranges indicating remission/relapse may vary depending on multiple factors, e.g. the used test kits, patient ethnicity/age, and physician experience Bjarnason (2017). For instance, fCP concentration between 50 and 150 μg/g could be considered insufficient to assess IBD remission and the test might need to be repeated. In our study, 6 patients (two from the annotated data subset) had fCP levels in the 50-150 μg/g range. In our analysis, we considered patients with fCP concentration above 250 μg/g having active inflammation. In the case of CRP, the biomarker concentration rapidly changes depending on acute inflammation and has shorter half time compared to other biomarkers Vermeire et al. (2006). Furthermore, CRP concentration changes highly depend on IBD disease, e.g. CRP response is more evident in CD than in UC cases Vermeire et al. (2006). Therefore, we hypothesised that BS acoustic features and CRP levels could change at a different pace over time when there is active inflammation.

3.3 IBD Type Classification

GBC was retrained on the patient group only to evaluate class separability between UC and CD. EffUNet annotations were used to extract features from BS events. The recordings were split into classification windows of duration δ=10 min, yielding to a dataset of 682 classification windows (273 from UC and 409 from CD patients respectively). Stratified group 10 fold CV was used to train and evaluate the model on the features selected at Sec. 3.2. A mean AUROC of 0.47 (std=0.09) and a mean accuracy of 0.44 were achieved. In particular, most classification windows Si were classified as belonging to CD patients. IBD type diagnosis can be challenging even with traditional assessments, and possibly leads to the temporary diagnosis IBD unclassified (indeterminatecolitis) Guindi and Riddell (2004).

3.4 GBC Noise Test

We further investigated the specificity of the GBC model trained for the classification of IBD patients vs. healthy controls on non-BS audio data, i.e. noise audio segments. The noise test was performed to analyse if the GBC model did learn unwanted non-BS sound patterns, e.g. environmental sounds, instead of the actual BS patterns. We randomly sampled noise audio segments n_i from the reference channel placed on

the esophagus (see Fig. S1) of each study participant. The esophagus audio data clearly did not contain BS and thus was ideally suited for the noise test. Noise segments were sampled according to the audio segment duration distribution of the expert-annotated BS.

Noise segments n_i were preprocessed following the same approach as BS event processing: We applied a bandpass 8th Butterworth filter with bandwidth 60-5000 Hz. From each sampled noise segment n_i, we extracted the same features as used to train the GBC on the classification windows S_i, i.e. 11 MFCCs. Following the procedure of BS event processing, noise events that were sampled from the same 10 min classification window N_i, were grouped together to estimate feature statistics, i.e. feature mean was derived from the feature distribution.

The GBC model was left unmodified, i.e. the models used to derive performance results for IBD patients vs. healthy controls, was tested on classification windows N_i of the noise audio segments. The same Group-K-Fold Cross-Validation (CV) approach as used for IBD patients vs. healthy controls was applied, where the dataset was split into K groups. For noise tests on the annotated data subset and the full dataset, 9-fold and 21-fold CV configurations were chosen, respectively. GBC performance was evaluated across all K CV folds by computing the AUROC, sensitivity, and specificity. Performance statistics were calculated from the results of all testing folds.

Figure S4 shows the GBC noise test results. Overall, the GBC reached a mean AUROC of 0.60 across all experiments, regardless of the BS retrieval method employed. The AUROC results suggest that the GBC models were unable to classify the noise segments into patient vs. healthy categories. Thus, our noise test indicates that the GBC could detect IBD based on relevant audio information derived from BS features, rather than environmental noise properties. However, some CV folds achieved AUROC above 0.60 when using the EffUNet-retrieved BS instances (see Fig. S4b-c). We hypothesised that the increased performance of some CV folds could have been caused by false positives of the EffUNet BS spotting stage. During the EffUNet training, ambient noise crosstalk into the BS training instances could not be fully excluded. Since in two dataset configurations, GBC was trained on the retrieved BS instances, some noise data may have affected the GBC training data. Our assumption is supported by the partially overlapping feature distributions of EffUNet-retrieved BS events and random noise segments n_i. Yet, the extend of the noise effect on the GBC models remained negligible.

REFERENCES

Bjarnason, I. (2017). The Use of Fecal Calprotectin in Inflammatory Bowel Disease. *Gastroenterology & Hepatology* 13, 53–56

De Cheveigné, A. and Kawahara, H. (2002). Yin, a fundamental frequency estimator for speech and music. *The Journal of the Acoustical Society of America* 111, 1917–1930

[Dataset] Genesis1 (2020). Digestive-system - 3d model by genesis1. Accessed: 6 July 2020

Gouyon, F. and Herrera, P. (2001). Exploration of techniques for automatic labeling of audio drum tracks instruments. In *Proceedings of MOSART: Workshop on Current Directions in Computer Music*

Guindi, M. and Riddell, R. H. (2004). Indeterminate colitis. *Journal of Clinical Pathology* 57, 1233–1244. doi:10.1136/jcp.2003.015214

Plomp, R. and Levelt, W. J. M. (1965). Tonal consonance and critical bandwidth. *The journal of the Acoustical Society of America* 38, 548–560

Vermeire, S., Van Assche, G., and Rutgeerts, P. (2006). Laboratory markers in IBD: Useful, magic, or unnecessary toys? *Gut* 55, 426–431. doi:10.1136/gut.2005.069476
